# Schockmed Valve: A Novel Surgical Option for Uncontrolled Glaucoma in Eyes with Poor Conjunctiva and Encircling Bands

**DOI:** 10.5005/jp-journals-10028-1236

**Published:** 2017-10-27

**Authors:** David Fleischman, Bryan Kim

**Affiliations:** 1 Assistant Professor, Department of Ophthalmology, University of North Carolina at Chapel Hill, Chapel Hill, North Carolina, USA; 2 Private Practitioner, Department of Ophthalmology, Everett Clinic, Everett Washington, District of Columbia, USA

**Keywords:** Ahmed valve, Encircling band, Glaucoma surgery, Schocket, Scleral buckle.

## Abstract

Surgical management of elevated intraocular pressure (IOP) in an eye with scarred conjunctiva, an encircling band, and silicone oil has limited options. By combining the flow restrictor of the Ahmed Glaucoma Valve (New World Medical, Rancho Cucamonga, California) and the capsule of the encircling band as a conduit for aqueous flow, immediate pressure reduction could theoretically be achieved in eyes with limited conjunctival mobilization. This is a description of a surgical technique in a case of a patient with multiple ocular surgeries with uncontrolled multimechanism glaucoma despite using all available topical and oral glaucoma medications. A combination of the Ahmed valve with a modification of the Schocket shunt technique was used in this case of an encircling band and poor conjunctiva. At 10-month follow-up, the patient maintains controlled pressures on dorzolamide-timolol drops. The combined Ahmed-Schocket (or Schockmed) technique may be another surgical option for management of uncontrolled IOP in cases of scarred conjunctiva and encircling bands needing immediate pressure reduction.

**Schockmed valve:**

A novel surgical option for uncontrolled glaucoma in eyes with poor conjunctiva and encircling bands.

**How to cite this article:** Fleischman D, Kim B. Schockmed Valve: A Novel Surgical Option for Uncontrolled Glaucoma in Eyes with Poor Conjunctiva and Encircling Bands. J Curr Glaucoma Pract 2017;11(3):120-124.

## INTRODUCTION

Eyes that have undergone prior surgery involving the conjunctiva pose a difficult challenge to the glaucoma surgeon. Limbal peritomy and other violations of the conjunctiva can lead to scarring and decreased conjunctival mobility. Creation of subconjunctival potential spaces and surgical closure of conjunctival wounds in these eyes can be difficult to perform. The scleral buckle is an important tool in the treatment of retinal detachment, but requires significant conjunctival dissection during its implantation. If these eyes subsequently develop glaucoma or refractory elevated IOP, the management options can become limited. The options become even more limited if the eye is filled with silicone oil.

Cyclodestructive procedures are useful, but can have a negative effect on visual acuity. Due to the poor state of the conjunctiva after multiple surgeries, and because of the need for inferior surgical placement in cases with silicone oil, trabeculectomy is often not a plausible option. Glaucoma tube shunt devices, such as the BAERVELDT Glaucoma Implant (Abbott Medical Optics, Inc, Santa Ana, California) and the Ahmed Glaucoma Valve are better suited for inferior placement. However, scleral buckles limit the amount of space available to implant the glaucoma tube shunt while still maintaining a safe distance from the limbus to prevent tube and plate migration and exposure.

Herein, we describe a novel surgical approach in the case of a patient who required immediate pressure correction, but with limited space and conjunctival mobility due to numerous prior surgeries.

## MATERIALS AND METHODS

### History

A 60-year-old White female underwent complicated cataract phacoemulsification with dropped lens fragments, and subsequently developed a retinal detachment. The patient had the lenticular material removed and the retinal detachment repaired. The patient then developed proliferative vitreoretinopathy, redetached, and had two more vitreoretinal surgeries including the placement of silicone oil and a scleral buckle. Four months after the surgery, the IOP began to increase. Gonioscopically, there was no evidence of blood in Schlemm’s canal nor silicone oil in the angle, and was overall open to scleral spur without peripheral anterior synechiae. The IOP was consistently in the high 30 to low 40 range despite maximal medical therapy, including 500 mg of oral acetazolamide extended release twice daily.

**Figs 1A to D:**
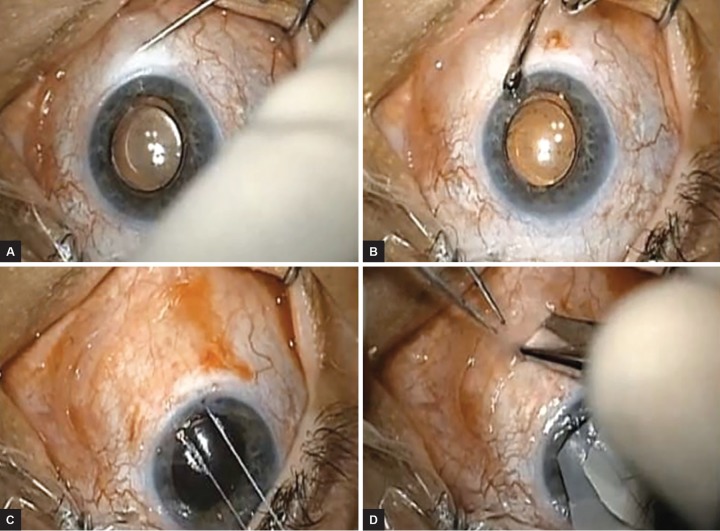
(A) Subconjunctival injection of lidocaine on a 30-gauge needle; (B) A muscle hook is used to distribute the subconjunctival lidocaine; (C) A traction suture is placed and the eye is supraducted; and (D) Conjunctival and tenons dissection performed approximately 5 mm posterior to the limbus

### Surgical Technique

In the case of intraocular silicone oil, an inferior tube placement is warranted to prevent occlusion of the tube by oil.^1^ The conjunctiva was inspected with two Weck-Cel sponges to identify where conjunctival mobilization seemed possible. Subconjunctival lidocaine with epinephrine was injected through a 30-gauge needle to dissect and elevate the conjunctiva and then was distributed with a muscle hook to expand plane dissection (Figs 1A to 1C).

**Figs 2A to D:**
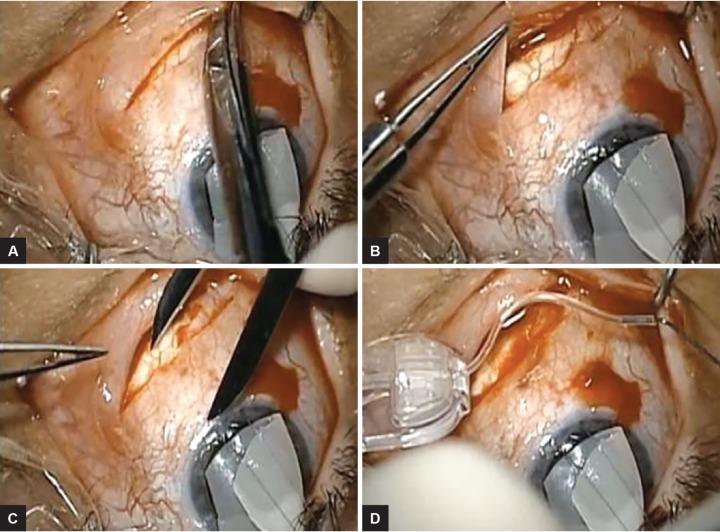
(A) The dissection is extended to just over 2 clock hours; (B) Posterior and capsular dissections to the scleral buckle; (C) Calipers used to identify 8.0 mm posterior from the limbus, which is then cauterized as a landmark. The anterior aspect of the plate must not extend anterior; and (D) The Ahmed Glaucoma Valve is primed

A modified fornix-based incision was used. Dissection posteriorly under conjunctiva and Tenon’s layer toward the scleral buckle was performed. There was a very thin layer overlying the capsule, and dissection was rather easy to accomplish. Calipers were used to measure the distance posteriorly to ensure that the glaucoma tube shunt will sit at least 7 to 8 mm behind the limbus, and, in this case, the buckle was at least 10 to 11 mm posterior to the limbus, allowing sufficient room for placement of the modified valve. The Ahmed Glaucoma Valve FP7 was brought onto the field and primed (Figs 1D and 2). The plate was trimmed from the valve, leaving only a small ridge of plate intact and the aqueous pool. This remaining valve and plate were placed on the field to ensure that its most anterior aspect was at least 7 to 8 mm posterior to the limbus. The valve is placed aside carefully. The capsule of the scleral buckle was dissected to the horizontal length of the valve, and a cyclodialysis spatula was used to create a plane between the buckle and capsule, both superiorly and laterally. This dissection is different than a standard Schocket procedure in which only one direction is utilized.

**Figs 3A to D:**
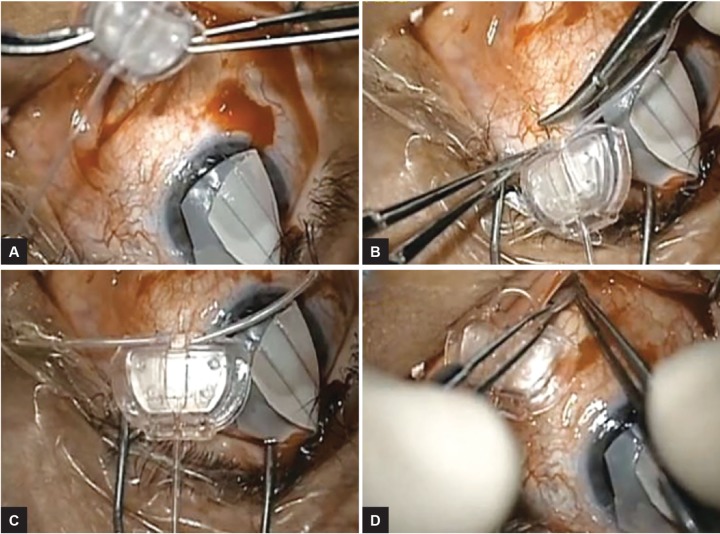
(A) The plate of the Ahmed Glaucoma Valve is cut with Stevens scissors, only leaving a small ridge and the aqueous pool of the valve; (B) Crawford nasolacrimal tubing is cut into two pieces, beveled, and sutured to the ridge facing the aqueous pool with 9-0 nylon through the lumen; (C) Final appearance of the Schockmed valve; and (D) After creating a plane between the capsule and the scleral buckle with a cyclodialysis spatula, both ends of the tubing are tucked along the tracts of the scleral buckle

**Figs 4A to D:**
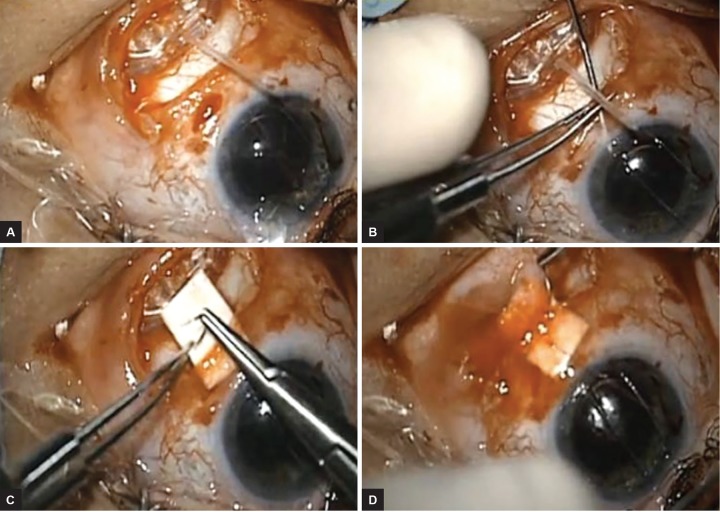
(A) The Schockmed valve is placed firmly against the scleral buckle, again ensuring the anterior aspect of the valve is at least 8.0 mm posterior to the limbus, and secured to the sclera with 9-0 nylon; (B) Sclerotomy with a 23-gauge needle; (C) Patch graft secured to the sclera with 7-0 vicryl; and (D) Conjunctival closure with 8-0 vicryl

Crawford silicone tubing was brought onto the field and cut into two 8 to 10-mm sections. Each end of each tube was beveled. The tube was then sutured with 9-0 nylon to the small ridge of plate at the edge of the valve (Figs 3A to C). The beveled ends of the tubes were introduced into the space created in the buckle-capsular complex, and the valve placed posteriorly until the edge of the valve and plate is abutting the buckle (Figs 3D and 4A). In doing so, the surface area of the aqueous pool has been increased by the direct interaction of the valve and buckle, as well as the extension provided by the tubing. The modified Ahmed Glaucoma Valve was sutured into place on the sclera with 9-0 nylon sutures with the knots rotated into the islets. The anterior chamber tube of the Ahmed device was cut to size with a bevel. The anterior chamber was entered with a 23-gauge needle, and the tube is introduced into the anterior chamber (Figs 4B to D). A patch graft was placed over the tube and tube entry site. If conjunctival mobilization was further limited, a native scleral flap or a scleral tunnel as per Albis-Donado et al^2^ could have been created to cover the tube, but this was not needed in this case. The conjunctival wound was closed with 8-0 vicryl suture.

**Fig. 5:**
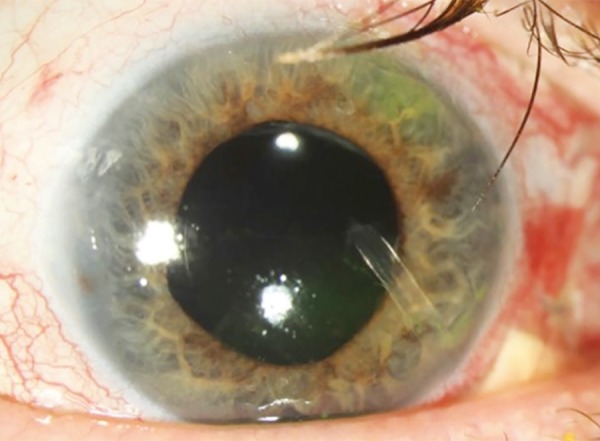
Postoperative visit week 1 with an inferonasal Schockmed valve in an eye with silicone oil and an encircling band

## RESULTS

The patient had immediate pressure control, with nearly all readings under 20 mm Hg (Fig. 5). Four months after surgery, pressure had increased to 20 mm Hg, and the patient was started on dorzolamide-timolol drops, twice daily in the operative eye. Her pressures have seen maintained in the mid-to-high teens. Her visual acuity remains stable (20/60). Six months after surgery, she had silicone oil removed from the eye by the vitreoretinal service, and pressures remained controlled. Some silicone oil has cuffed around the tube in the anterior chamber, but has not occluded the tube, and there is no evidence of bleb or subconjunctival migration.

## DISCUSSION

Secondary glaucoma after complex retinal detachment surgery is common and creates a challenge for the retina and glaucoma surgeon. There are likely numerous mechanisms that contribute to the development of elevated IOP, such as angle closure from ciliary body congestion from scleral buckling, silicone oil migration into the anterior chamber, Schwartz’s syndrome, and many others.^3,4^

The Schocket procedure, or the anterior chamber tube to scleral encircling band, is a useful procedure for IOP management in eyes with an encircling band.^5^ However, in many of these cases, the eyes have had numerous prior surgeries, and, therefore, conjunctival mobilization is limited, making the large dissection spanning both ends of an intraocular muscle less desirable. Additionally, the Schocket is not valved and, therefore, typically needs to be ligated to allow further encapsulation prior to the opening of the tube. This results in a 4- to 6-week continuation of increased IOP. Fenestration of the tube may be used to temporize IOP, but the results of fenestration vary significantly and rarely last until release of the ligature.

The Ahmed glaucoma valve, with its flow restrictor, is useful in cases requiring immediate IOP reduction. However, due to the size of its plate, the device cannot be used in its entirety in eyes with an encircling buckle. By trimming the excess plate off of the valve, the valve can be made to fit the reduced space created by the presence of the encircling buckle. The decreased surface area of the trimmed plate, however, may limit the degree of IOP reduction. This problem is addressed by incorporating the capsule of the encircling band though use of the Schocket tubes. As a result, the surface area available to the modified Ahmed valve for aqueous diffusion is greatly increased. Therefore, combining the principles of the Schocket tube to maximize the surface area of the Ahmed valve, while minimizing the amount of conjunc-tival dissection by utilizing both the anterior and lateral extensions of the buckle may be a solution to these difficult cases.

## References

[1] Friberg TR, Fanous MM (2004). Migration of intravitreal silicone oil through a Baerveldt tube into the subconjunctival space. Semin Ophthalmol.

[2] Albis-Donado O, Gil-Carrasco F, Romero-Quijada R, Thomas R (2010). Evaluation of Ahmed glaucoma valve implantation through a needle-generated scleral tunnel in Mexican children with glaucoma. Indian J Ophthalmol.

[3] Budenz DL, Taba KE, Feuer WJ, Eliezer R, Cousins S, Henderer J, Flynn HW Jr (2001). Surgical management of secondary glaucoma after pars plana vitrectomy and silicone oil injection for complex retinal detachment. Ophthalmology.

[4] Gedde SJ (2002). Management of glaucoma after retinal detachment surgery. Curr Opin Ophthalmol.

[5] Schocket SS (1986). Investigations of the reasons for success and failure in the anterior shunt-to-the-encircling-band procedure in the treatment of refractory glaucoma. Trans Am Ophthalmol Soc.

